# Supplementation with conjugated linoeic acid decreases pig back fat deposition by inducing adipocyte apoptosis

**DOI:** 10.1186/1746-6148-10-141

**Published:** 2014-06-26

**Authors:** Renli Qi, Feiyun Yang, Jinxiu Huang, Han Peng, Yan Liu, Zuohua Liu

**Affiliations:** 1 Chongqing Academy of Animal Science, Rongchang, Chongqing 402460, China; 2 Key Laboratory of Pig Industry Sciences, Ministry of Agriculture, Rongchang, Chongqing 402460, China; 3 Chongqing Key Laboratory of Pig Industry Sciences, Rongchang, Chongqing 402460, China; 4 Southwest University, Chongqing 400715, China

**Keywords:** Conjugated linoleic acid, Fat deposition, Adipocyte, Apoptosis, Pig

## Abstract

**Background:**

Conjugated linoleic acid (CLA), a C18 fatty acid with conjugated double bonds, has been shown to serve as a powerful anti-obesity agent by several research groups, although the precise mechanism remains elusive. Previous studies showed that CLA induced apoptosis in 3T3-L1 cells and in mice. The aim of this research was to clarify the role of CLA in adipocyte apoptosis in pigs, a relevant model for obesity research.

**Results:**

Our results clearly show that back fat deposition of CLA-fed pigs was significantly lower than that of pigs in the control group. Moreover, some typical apoptotic cells were observed among the adipocytes of CLA-fed pigs. Furthermore, the CLA-fed pigs had reduced expression of the anti-apoptosis factor Bcl-2 and increased expression of the pro-apoptosis factors Bax and P53. Subsequently, increased cytochrome C was released from the mitochondria to the endochylema, and the caspase cascade was activated, resulting in cellular apoptosis. These results are consistent with the effects of Bcl-2 and Bax in regulating CLA-induced adipocyte apoptosis via the mitochondrial signaling pathway. However, the increased expression of tumor necrosis factor (TNF)-α and its receptor TNFR indicate that the effect of CLA might partly be through the death receptor signaling pathway in adipose cells.

**Conclusions:**

Our study has demonstrated that CLA reduces pig body fat deposition, an outcome that is partly meditated by apoptosis of adipose cells, and that both the mitochondrial pathway and the death receptor pathway are involved in this effect.

## Background

Conjugated linoleic acid (CLA) belongs to the family of 18 carbon fatty acids containing conjugated double bonds. It is found in dairy products and ruminant meats as a mixture of positional and geometric (Cis or Trans) isomers of linoleic acid (18:2 n-6). There is increasing evidence that CLA has powerful anti-adiposity functions in both humans and animals [1-3]. CLA treatment notably decreases body fat deposition and changes the route of fat metabolism, effects that have been observed in several different studies [2-6]. Although the precise mechanism is still unclear, results of these studies demonstrate that CLA may inhibit the proliferation and/or differentiation of adipose cells, and the synthesis and accumulation of triglycerides in adipocytes.

Recently, some research groups have shown that CLA can induce apoptosis of fat cells in mice and in 3T3-L1 adipocytes. In 2000, Tsuboyama-Kasaoka *et al.* first reported that supplying feed supplemented with 1% CLA to C57BL/6 J mice for 5 months resulted in a clear decrease in the abdominal fat pad of the mice, with the adipocytes exhibiting typical apoptotic characteristics, such as DNA fragmentation [7]. Subsequent studies showed that CLA also induced adipose cell apoptosis either *in vitro* or *in vivo*[8-11]. It has long been known that CLA has anti-cancer effects and can directly induce apoptosis of a number of different tumor cell types [12-15]. However, the effects of CLA on apoptosis in adipose cells have not been widely reported, and the molecular mechanisms by which these effects occur are still unclear.

The aim of the current study was to clarify the role of CLA in adipocyte apoptosis in pigs, whose physiological characteristics are similar to humans, thus making them a highly relevant model for obesity research. Additionally, the molecules that regulate apoptosis in adipocytes were investigated.

## Results

### CLA supplementation reduces back fat accumulation in pigs

In our experiment, piglets were fed with 0, 1%, or 2% CLA for 30 days. Both the 1% and 2% CLA-fed pigs demonstrated a loss of body weight compared with the control pigs by the end of the experiment. Back fat tissue and abdominal fat pads of the piglets were collected, weighed, and analyzed. The results showed that CLA-fed pigs had less body fat deposition, particularly of back fat (*P* < 0.05, Table 1). Additionally, CLA-fed pigs demonstrated an increased accumulation of intramuscular fat, indicating that CLA has tissue-based differential effects on fat metabolism.

**Table 1:** Body weight and body fat

**Items**	**Control**	**1% CLA**	**2% CLA**
Initial body weight, kg	13.91 ± 1.68	13.91 ± 1.55	13.91 ± 1.37
Finished body weight, kg	30.39 ± 1.36	28.10 ± 2.38	28.46 ± 1.47
Feed intake, kg	36.13 ± 2.77	30.96 ± 3.51	31.43 ± 2.27
Back fat weight, g	370.48 ± 33.63^A^	319.60 ± 17.64^B^	297.68 ± 14.32^B^
Abdominal fat weight, g	179.04 ± 27.70	172.24 ± 19.69	166.48 ± 9.35
Intramuscular fat in dorsal muscles, %	0.71 ± 0.27	0.71 ± 0.14	0.73 ± 0.17
Intramuscular fat in leg muscle, %	1.35 ± 0.38	1.37 ± 0.18	1.39 ± 0.34

Note: In the same row, values with different capital letter superscripts mean significant difference (P < 0.05).

### CLA supplementation induces apoptosis of adipose cells

**Figure 1:**
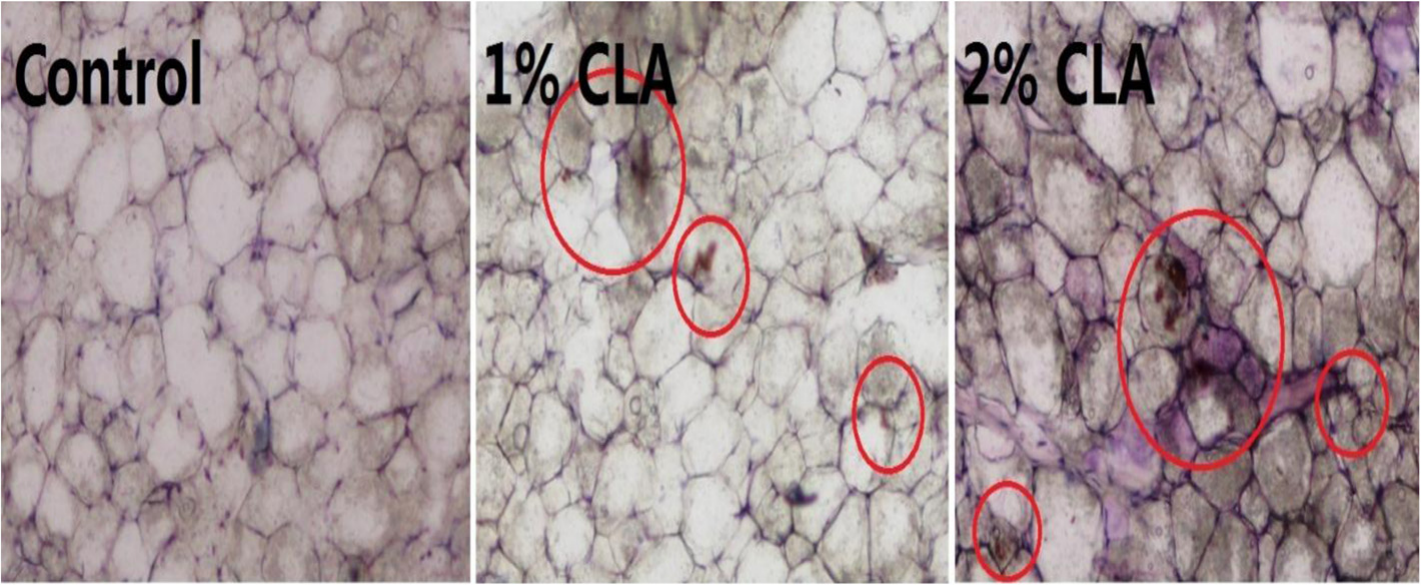
**TUNEL assay of apoptotic cells (200× magnification).** Pig back fat cryosections were analyzed using a TdT-mediated dUTP nick end labeling (TUNEL) apoptosis detection kit (Beyotime, China) according to the manufacturer’s instructions. Typical apoptotic cells (brown spots) are emphasized with red rings in this figure. This clearly shows that the 2% CLA-fed pigs had more apoptotic fat cells.

**Figure 2:**
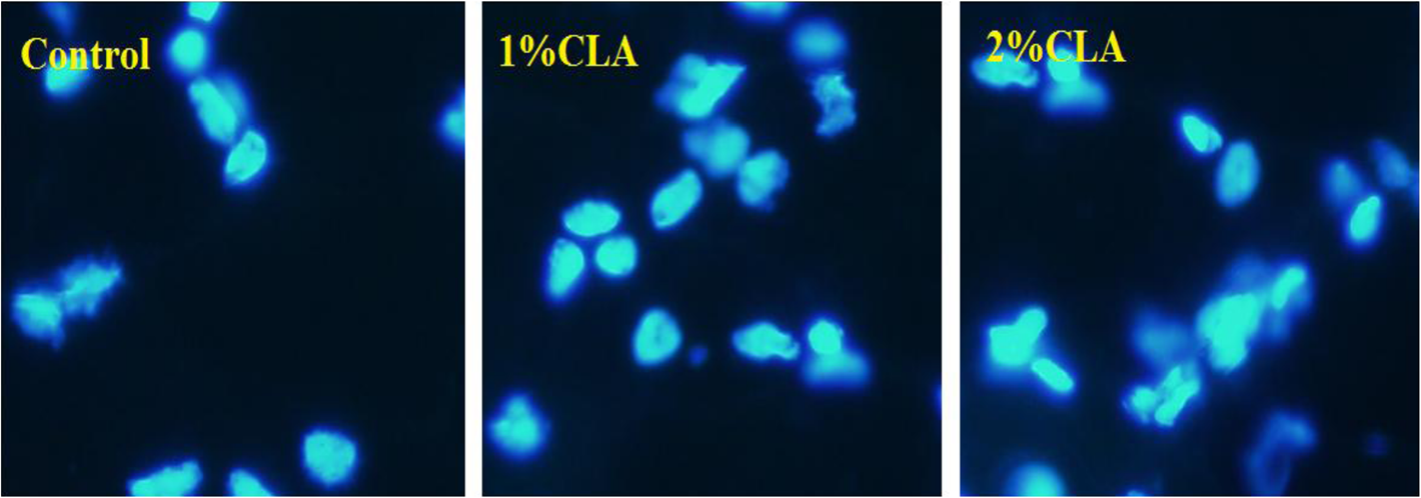
**Hoechst 33258 staining of back fat sections (100× magnification).** Pig back fat cryosections were fixed in 4% neutral formalin and stained with Hoechst 33258 (10 μg/mL). The number of apoptotic cells (i.e., those showing chromatin condensation and fluorescence enhancement) was assessed by observation with an inverted fluorescence microscope. There were more apoptotic cells present in the 2% CLA-fed pigs than in pigs in the other two groups.

**Figure 3:**
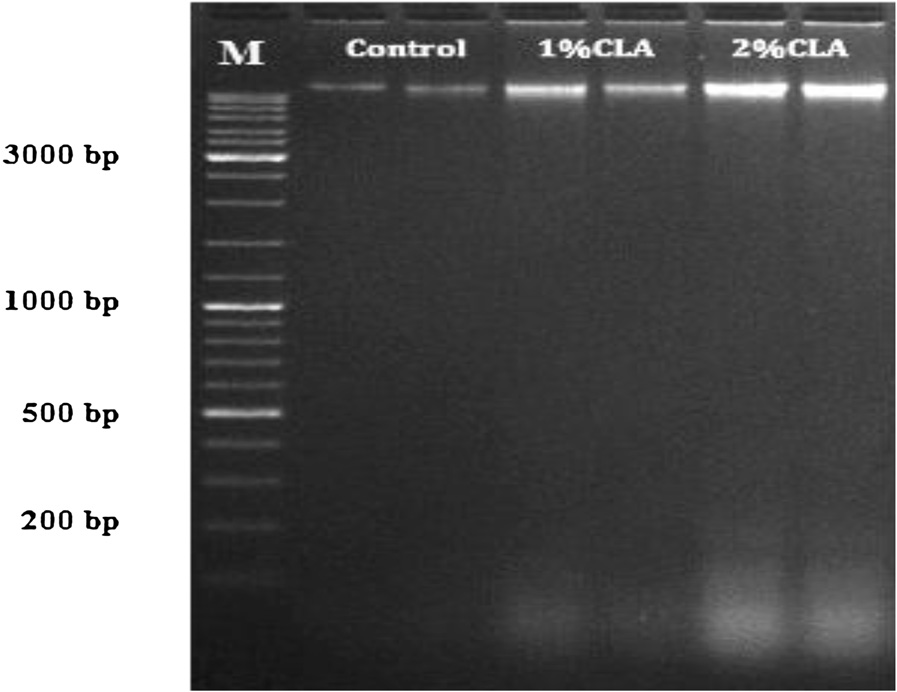
**Agarose gel electrophoresis of DNA fragments in back fat samples from piglets.** Genomic DNA from the fat tissue was extracted, and assessed using agarose gel electrophoresis with ethidium bromide. M stands for the DNA marker. Compared with control piglets, the 2% CLA treatment showed increased numbers of DNA fragments (length less than 200 bp) in the back fat of piglets.

In the present study, some typical apoptotic phenomena such as cell shrinkage, chromatin condensation, fluorescence enhancement, and degradation of DNA in cellular chromatin were observed in the adipose cells of CLA-fed pigs (particularly 2% CLA-fed pigs) using the TUNEL assay (Figure 1), Hoechst 33258 staining (Figure 2), and agarose gel electrophoresis of DNA (Figure 3). These results indicate that CLA supplementation induces and/or promotes apoptosis of pig adipocytes.

### Mitochondrial signaling pathway plays an important role in CLA-induced apoptosis

**Figure 4:**
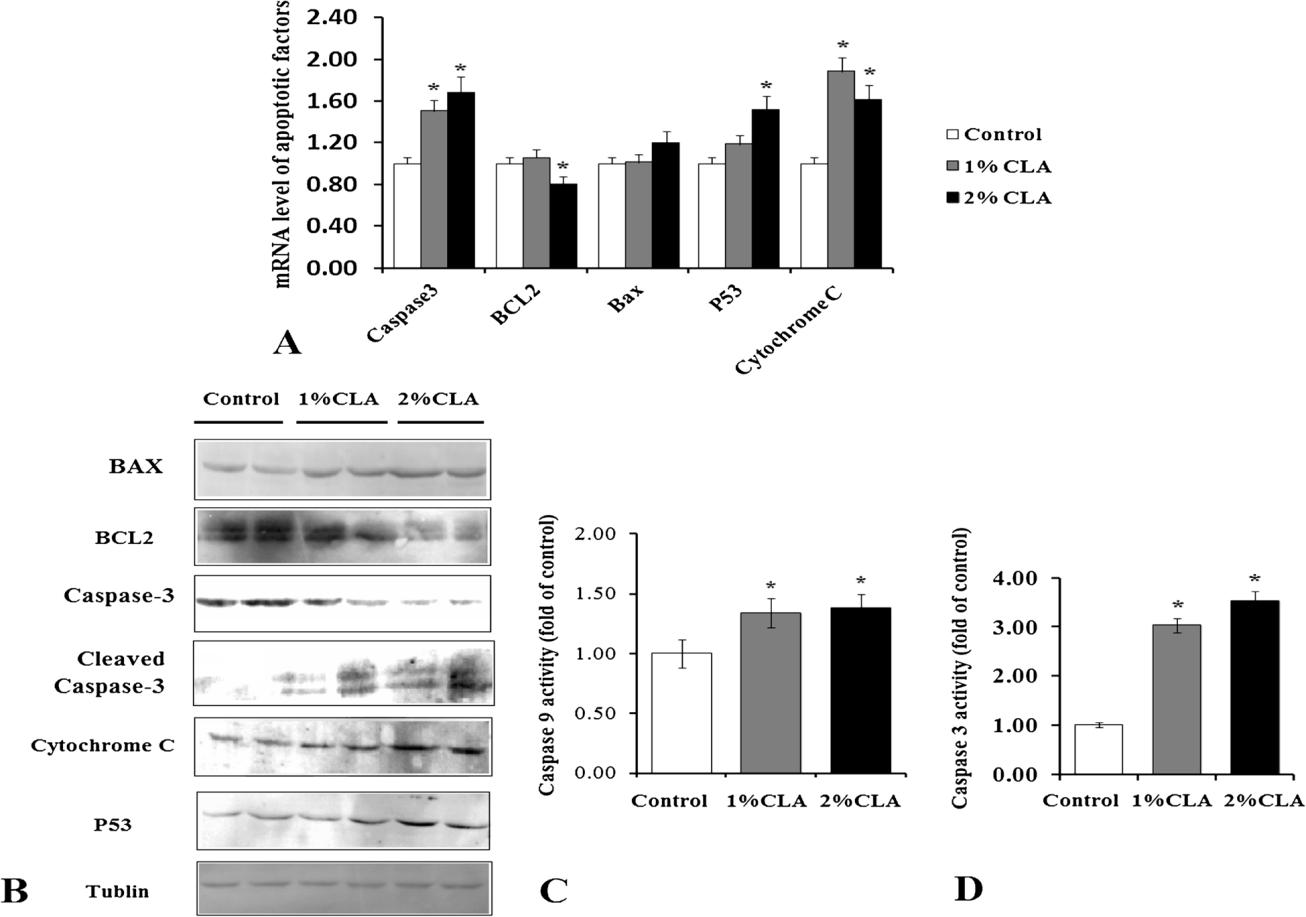
**Expression and activity changes of apoptotic regulators. A**. mRNA levels of apoptotic regulators in back fat of pigs. mRNA levels were measured using RT-qPCR. **B**. Protein concentrations of apoptotic regulators in back fat of pigs. **C**. Caspase-9 activity in back fat of pigs. **D**. Caspase-3 activity in back fat of pigs. data are shown as fold-change compared with control. n = 5, *P < 0.05 *vs.* Control.

A number of molecules and signal transduction pathways participate in the apoptotic process. Back fat tissue of CLA-fed pigs had lower expression of Bcl-2, a key inhibitor of cellular apoptosis, at both mRNA (Figure 4A) and protein (Figure 4B) levels. Furthermore, mRNA and protein expression of the pro-apoptotic molecule Bax were slightly increased in the back fat of CLA-fed pigs (Figure 4A and B). These two factors function in the early part of the mitochondrial signaling pathway, and changes in their expression initiate activation, and significantly increase the expression, of P53, as well as the release of cytochrome c to the cytoplasm (Figure 4A and B). Activity of caspase-9 showed a corresponding increase to 1.4 fold (Figure 4C), and expression level and activity of caspase-3 (Figure 4A and D), a key apoptotic protein, both increased significantly. These results suggest that the mitochondrial signaling pathway plays a key role in CLA-induced apoptosis of pig adipocytes.

### Death receptor signaling pathway trigged by tumor necrosis factor (TNF)-α also participates in CLA-induced apoptosis

**Figure 5:**
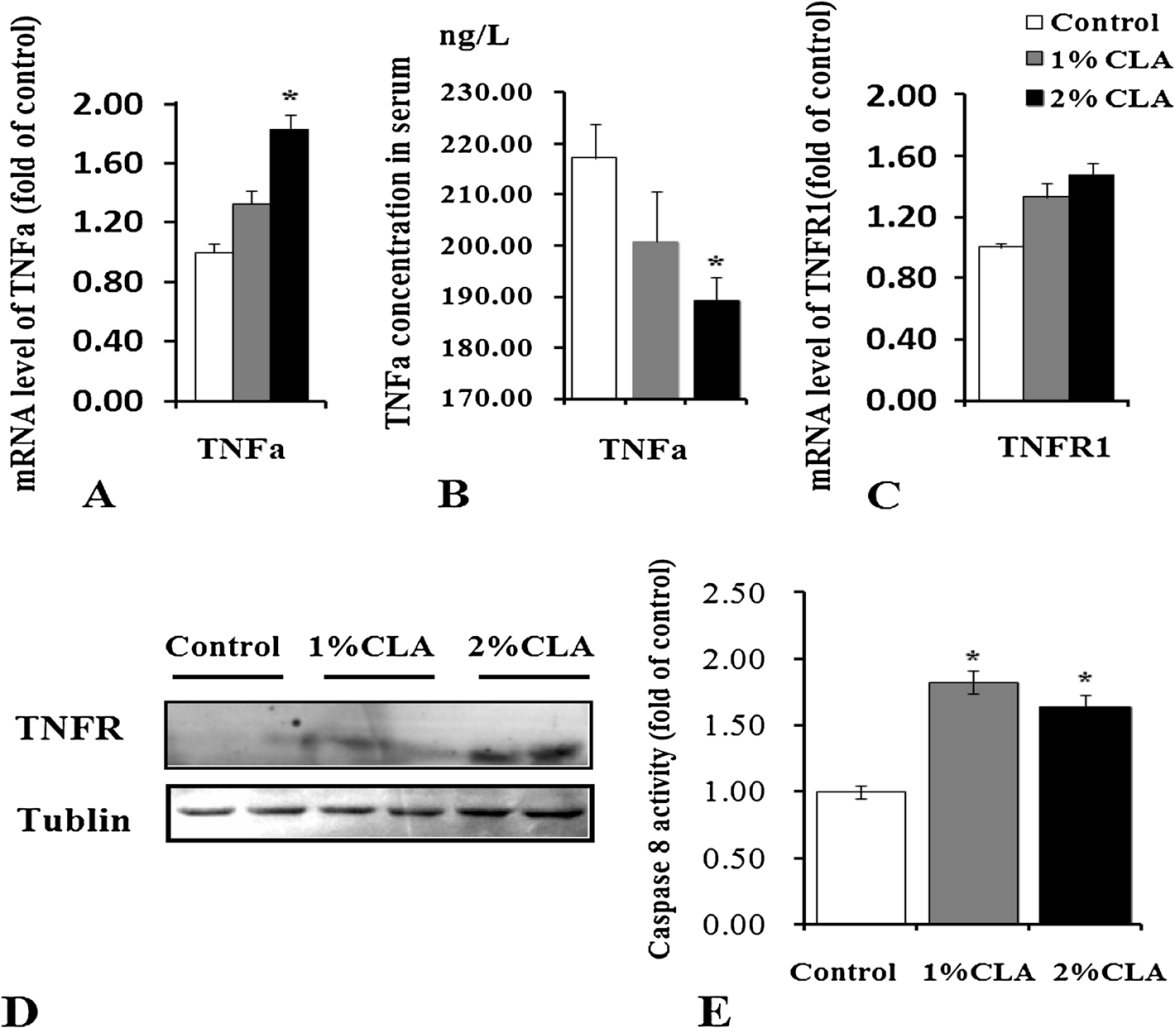
**TNF-α and TNFR expression and caspase-8 activity. A**. TNF-α mRNA in back fat of pigs. **B**. Serum TNF-α in pigs, **C**. TNFR mRNA in back fat of pigs, **D**. TNFR protein levels in back fat of pigs. **E**. Caspase-8 activity in back fat of pigs. n = 5, *P < 0.05 *vs.* Control.

We also examined the mRNA level and the circulating concentration of TNF-α, an inflammatory factor and apoptosis inducer secreted by adipocytes. A previous study reported that TNF-α could induce cellular apoptosis as a ligand that activates the death receptor signaling pathway [16]. Compared with control pigs, 2% CLA–fed pigs showed significantly increased TNF-α mRNA expression (Figure 5A) in their back fat tissue (*P* < 0.05). However, the circulating concentration of TNF-α protein (Figure 5B) was significantly decreased (*P* < 0.05). Therefore, we speculate that a high level of TNF-α in back fat might play a role in the induction of adipocyte apoptosis by CLA. Furthermore, higher expression levels of TNFR (Figure 5C and D) and higher activity of caspase-8 (Figure 5E) in the fat tissue of CLA-fed pigs indirectly support this suggestion.

## Discussion

Fat metabolism and its related syndromes have been a primary focus of scientific research for many years. An increasing number of agents have been discovered for treating obesity, hypertension, hyperlipidemia, and type 2 diabetes [17,18]. In recent years, CLA, because of its robust anti-obesity effects, has gained increasing attention in studies on fat metabolism. Our results are in agreement with previous studies showing that young pigs administered CLA-supplemented feeds have less body fat (particularly back fat).

The apoptotic process is characterized by programmed cell death regulated by numerous genes [19]. Owing to the characteristics of adipocytes (a large amount of intracellular lipid droplets and a consequent difficulty to form apoptotic bodies), apoptosis research in adipocytes has been less than that in other cell types. However, some recent studies have led to the belief that there is no obvious difference between apoptosis in adipocytes and apoptosis in other cells [20,21]. CLA has long been considered an effective anti-cancer drug because it can induce apoptosis in various tumor cells and/or enhance the cells’ susceptibility to apoptosis. Very few papers have reported that CLA can also induce adipocyte apoptosis in rodents, or in adipocyte cell lines [7-10]. To investigate whether CLA also induces apoptosis in pig adipocytes, the key aim of the current study, we examined (and detected typical apoptotic phenomena in adipose cells from the CLA-fed pigs (particularly in the 2% CLA-fed pigs), as we had expected. We therefore have reason to believe that CLA can exert its anti-obesity function, at least partly, via inducing apoptosis in adipose cells.

**Figure 6:**
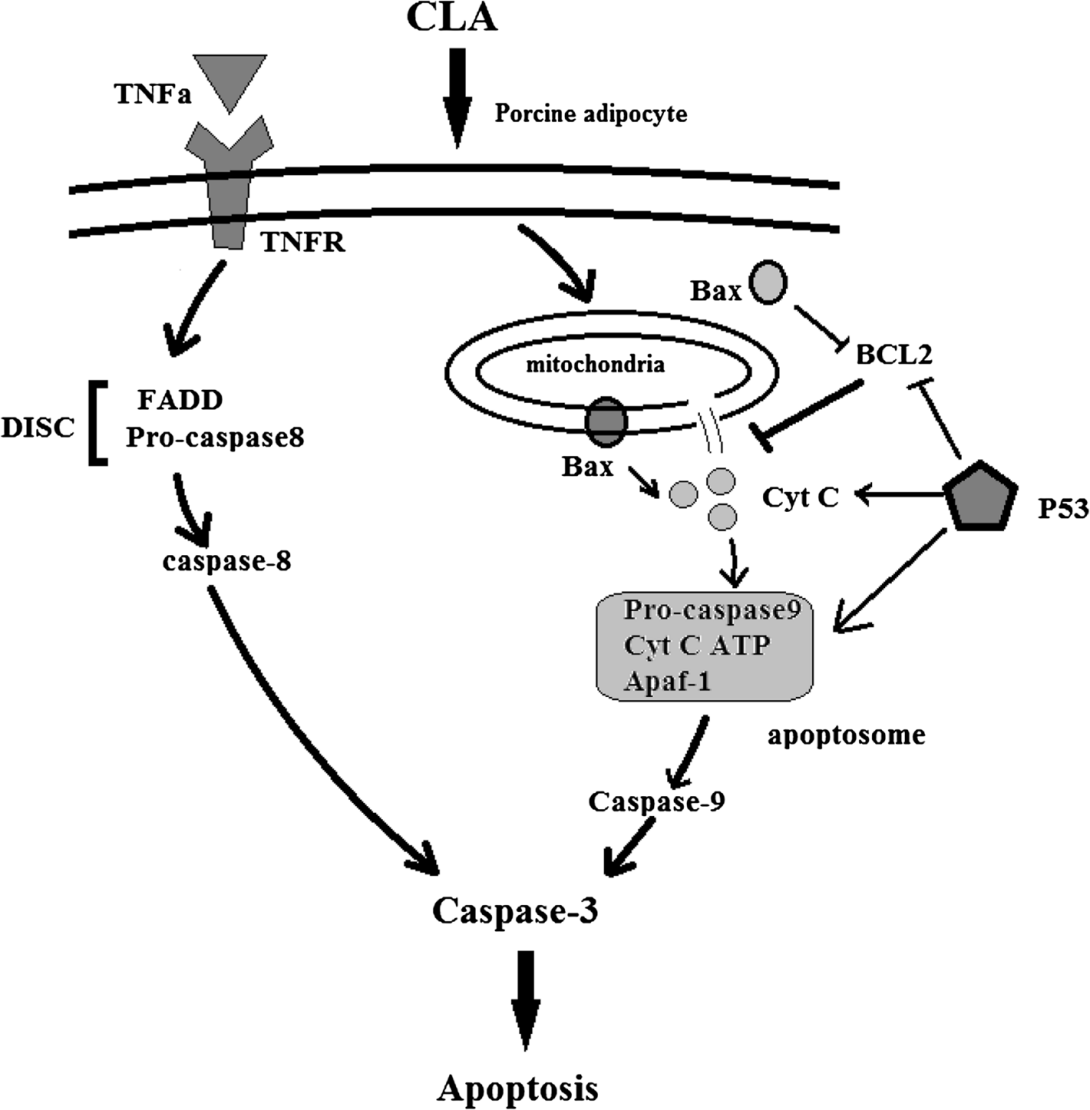
Apoptotic signal transduction in adipocytes induced by conjugated linoleic acid (CLA).

The mechanisms of apoptosis are highly complex and sophisticated, involving an energy-dependent cascade of molecular events [19]. To date, research indicates that there are two main apoptotic pathways, the extrinsic or death receptor pathway and the intrinsic or mitochondrial pathway [19,22]. The extrinsic and intrinsic pathways converge on the same execution pathway. This pathway is initiated by the cleavage of caspase-3 and results in DNA fragmentation, degradation of cytoskeletal and nuclear proteins, cross-linking of proteins, formation of apoptotic bodies, and finally uptake by phagocytic cells [23]. Changes in both expression and activity of key apoptosis factors in the current experiment suggest that both the intrinsic and extrinsic apoptotic pathways were activated in the fat tissue of CLA-fed pigs (Figure 6). In fact, there is recent evidence showing that the two pathways are linked, and that molecules in one pathway can influence the other [24].

In the intrinsic pathway, stimuli such as drugs, radiation, or hypoxia induce DNA injury, change mitochondrial permeability, and then discharge more active apoptotic molecules such as cytochrome C, which then combine with apoptotic protease activating factor-1 (Apaf-1) and procaspase-9 to form an apoptosome. In this complex, self-cleavage and activation of procaspase-9 generates caspase-9, which in turn activates caspase-3 in the presence of ATP. In this process, the Bcl-2 protein family plays a critical role. The family includes many proteins that are categorized based on their function as either pro-apoptotic proteins (Bcl-2, Bcl-xl, and Bcl-w) or anti-apoptotic proteins (Bax, Bak, Bad, and Bid) [25,26]. Bcl-2 inhibits the release of cytochrome C and inhibits the function of activated caspases while Bax has the opposite effect. P53 is a transcription factor that is often seen as an important pro-apoptotic factor because DNA-injury-increased P53 induces the expression of Bax and promotes the release of cytochrome C [27,28]. Based on the changes in the expression and activity of Bcl-2, Bax, cytochrome C, P53, and caspase-9, our results suggest that the intrinsic pathway plays an important role in the CLA-induced apoptosis of adipocytes. Similar studies have also shown the importance of the intrinsic pathway in this process.

In the extrinsic pathway, death factors (such as TNF-α) function as ligands that bind to their respective receptor proteins (for example, TNFR is a receptor for TNF-α), which then induces Fas-associated with death domain protein to combine with procaspase-8 to form a death-inducing signaling complex (DISC). Self-cleavage and activation of procaspase-8 in the DISC generates caspase-8 and subsequently activates caspase-3 [29]. TNF-α is a powerful pro-apoptotic factor secreted by fat in mammals [30]. Tsuboyama-Kasaoka *et al*. observed a six-fold increase of *Tnfa* mRNA level in 1% CLA-fed mice, and suggested that the increase was related to adipocyte apoptosis and decreased fat deposition [7]. In our studies, the increased expression of TNF-α, TNFR and the increased activity of caspase-8 demonstrate that the extrinsic pathway also participates in adipocyte apoptosis induced by CLA.

## Conclusion

As a potent anti-obesity agent, CLA has gained a lot of attention and its mechanism of action is gradually becoming clear. Though some studies have demonstrated that the inhibition of differentiation of adipocytes is the main mechanism by which CLA induces anti-obesity effects, we believe that CLA decreases body fat deposition in pigs at least partly by inducing adipocyte apoptosis. Furthermore, both the extrinsic pathway and the intrinsic pathway participate in this event.

## Methods

### Ethics statement

The present study was approved by the Ethics Committee of Chongqing Academy of Animal Science (Approval No. 2013-622), and the animal euthanasia and sample collection were in strict accordance with the requirements of the Ethics Procedures and Guidelines of the People’s Republic of China.

### Animal and sample collection

The experimental animals were fed in a standardized commercial pig farm with a spacious and clean housing. Forty-eight healthy pigs (Duroc × landrace × yorkshire, male) of approximately 14 kg body weight were randomly assigned to three groups (n = 16 in each group): a control group (0 CLA), a 1% CLA group, and a 2% CLA group, and their feed was supplemented with the corresponding level of a CLA mixture (c9, t11-CLA: t10, c12-CLA = 1:1, purchased from Aohai Biotechnology Co, Ltd. Qingdao, China). The experiment was conducted for 30 days, and food and water were offered to the animals *ad libitum* during this period. The diet formulation met the Chinese meat-fat type pig feeding standard (NY/T 95-2004).

All the piglets were weighed on days 1, 15, and 30 of the study. After weighing on days 15 and 30, five randomly selected piglets from each group were bled and then euthanized. The blood was collected to obtain plasma by centrifugation at 1,500 × *g* for 15 min. The back fat tissue and the abdominal fat pad were collected for frozen sections, RNA, DNA, and protein extraction. These tissue samples were snap frozen in liquid nitrogen as soon as they were obtained and stored at −80°C until analysis.

### DNA laddering detection

DNA was extracted from the fat tissue samples using a standard phenol/chloroform/isoamyl alcohol technique. The extracted DNA was separated on a 2% agarose gel containing 0.5 g/mL ethidium bromide and visualized on an ultraviolet transilluminator and photographed. Fragmented DNA appeared as a DNA ladder on the gel, indicative of apoptotic cell death.

### TUNEL assay

Apoptotic cells can be detected by terminal deoxynucleotidyl transferase (TdT)-mediated dUTP nick end labeling (TUNEL). The fat tissue of pigs was assayed using a colorimetric TUNEL Apoptosis Assay Kit (Beyotime, Beijing, China) according to the manufacturer's protocol. Frozen sections of 5 μm thickness were incubated with H_2_O_2_ (0.3% H_2_O_2_ in methanol) at room temperature (RT) for 30 min. After rinsing with 0.01 mol/L PBS, the sections were treated with biotin-dUTP in TdT reaction buffer at 37°C for 60 min followed by incubation with streptavidin-HRP at RT, and then rinsed twice with PBS. Finally, the sections were stained with DAB and observed under light microscopy.

### Hoechst 33258 staining

Frozen fat sections (5 μm thickness) were fixed for 10 min in 4% formaldehyde solution at 4°C. Following a rinse in PBS, the sections were incubated with an aqueous solution of Hoechst 33258 (10 μg/mL, Sigmaaldrich, China) at RT in the dark for 20 min. The sections were then rinsed again with water and evaluated by fluorescence microscopy (ultraviolet light, 340 nm).

### Analysis of the activity of caspase-3, 8, and 9

Activity levels of caspase-3, -8, and -9 in the fat tissue were measured using commercial assay kits (Beyotime, Beijing, China) according to the manufacturer’s instructions. Total protein was extracted from the fat tissue after grinding and subsequent lysing of the tissue in lysis buffer on ice. The protein concentration was measured using a bicinchoninic acid (BCA) Protein Assay Reagent (TianGen, Beijing, China). For the caspase-3 activity assay, equal amounts of total lysates were mixed with caspase-3 assay buffer containing the fluorogenic substrate Ac-DEVD- *p*NA (2 mM) in a 96-well plate in triplicate. Caspase-3 mediated cleavage of Ac-DEVD-*p*NA into free *p*NA (yellow) was measured using an excitation wavelength of 380 nm and emission wavelength of 460 nm, in a microplate reader (Gene Company Limited, Hong Kong, China). The fluorogenic substrates in the caspase-8 and caspase-9 activity assays were Ac-IETD-*p*NA (for caspase-8) and Ac-LEHD-*p*NA (for caspase-9), and the absorption wavelength used for both was 405 nm.

### Protein extraction and western blotting

The fat tissue extracts were prepared using 500 μL lysis buffer (Beyotime, Beijing, China) per 20 mg fat tissue, for 30 min at 4°C. Following centrifugation at 12,000 × *g* for 10 min at 4°C, the supernatants were removed and their protein concentrations determined using the BCA method. Total protein extracts were separated by 12% SDS-PAGE and transferred to polyvinylidene membranes (60 V for 4 h). The membranes were blocked with 5% nonfat milk in Tris-buffered saline containing 0.1% Tween 20 (TBST) at RT for 2 h, and probed overnight with primary antibodies at 4°C (anti-BCL2, anti-BAX, anti-cleaved caspase-3, anti-P53, anti-TNFR: Beyotime, Shanghai, China; anti-caspase3: ABcam, UK; anti-tubulin: Santa Cruz, USA). After washing with TBST, the membranes were probed with a horseradish peroxidase-labeled secondary antibody (1:10,000, Santa Cruz, USA) at RT for 1.5 h. Blots were visualized with a chemiluminescence reagent (Millipore, MA, USA) using an imaging system (BioRad, CA, USA).

### RNA extraction and RT-qPCR analysis

The mRNA levels of apoptosis regulators were measured using Real-time qPCR. PCR was performed using a StepOne system (ABI, NY, USA) using a 20-μL reaction mixture containing 12.5 μL SYBR *Premix Ex Taq*™ II (Takara, Dalian, China), 1 μL forward primer, 1 μL reverse primer, 2 μL template cDNA, and 8.5 μL ddH_2_O. The primer sequences are shown in Table 2. The cycling conditions consisted of an initial single cycle of 30 s at 95°C followed by 40 cycles of 5 s at 95°C and 35 s at 60°C. To correct differences in the amounts of template DNA, mRNA levels are shown as a ratio relative to the β-actin mRNA level.

**Table 2:** Primer sequences of qPCR

**Gene**	**Primer sequences (5′ → 3′)**
*BCL2*	F:TGATTTCTCCTGGCTGTCTC
	R:GCCCGTCCACTTCACTTAT
*Bax*	F:GAATGGGGGGAGAGACACCT
	R:CCGCCACTCGGAAAAAGA
Cytochrome C	F:GCCACCGCCTTATTTATTACAA
	R:GAAACATTCCATCAGCCATACA
*Caspase-3*	F:TCTAACTGGCAAACCCAAACTT
	R:AGTCCCACTGTCCGTCTCAAT
*P53*	F:TAGTTGCTGTTTCCGTGTGTTT
	R:GGCTTCCGACCCAGTGTAT
*TNFα*	F:CTCCCCTGTCCATCCCTTTAT
	R:CAGCCCCTCATTCTCTTTCTAA
*TNFR*	F:GCATTCTTCCTCTTCGTTGG
	R:GCTTGGGATGGGACTGAAAG
*β-actin*	F:GCGGCATCCACGAAACTAC
	R:TGATCTCCTTCTGCATCCTGTC

### Measurement of TNF-α concentration in serum

A commercial ELISA kit (R&D systems, USA) for porcine TNF-α was used to measure TNF-α concentration in the serum of piglets, according to the manufacturer’s instructions.

### Statistical analysis

Results are expressed as mean ± SEM, and comparisons between groups or within groups were made using one-way ANOVA, followed by Tukey’s Multiple Comparison Test. The Student’s t-test was used to determine significance when only two groups were compared, and *P* < 0.05 was considered statistically significant.

## Abbreviations

BCL-2: B-cell lymphoma-2; Caspase: Cysteinyl aspartate specific proteinase; CLA: Conjugated linoleic acid; TNF-α: Tumor necrosis factor α; TNFR: Tumor necrosis factor receptors.

## Competing interests

The authors declare that they have no competing interests.

## Authors’ contributions

RQ and ZL conceived the study and participated in its design. FY, JH, and HP carried out the animal experiment and molecular studies. YL performed the statistical analysis. All authors have read and approved the final manuscript.
